# Quantitative Relationships Between Growth, Differentiation, and Shape That Control *Drosophila* Eye Development and Its Variation

**DOI:** 10.3389/fcell.2021.681933

**Published:** 2021-07-19

**Authors:** Francisco Javier Lobo-Cabrera, Tomás Navarro, Antonella Iannini, Fernando Casares, Alejandro Cuetos

**Affiliations:** ^1^Department of Physical, Chemical and Natural Systems, Pablo de Olavide University, Sevilla, Spain; ^2^DMC2-GEM Unit, The CABD, CSIC-Pablo de Olavide University-JA, Seville, Spain

**Keywords:** organ growth, size, drosophila, eye development, mathematical modeling, IbM computational model, evolution, computer simulation

## Abstract

The size of organs is critical for their function and often a defining trait of a species. Still, how organs reach a species-specific size or how this size varies during evolution are problems not yet solved. Here, we have investigated the conditions that ensure growth termination, variation of final size and the stability of the process for developmental systems that grow and differentiate simultaneously. Specifically, we present a theoretical model for the development of the *Drosophila* eye, a system where a wave of differentiation sweeps across a growing primordium. This model, which describes the system in a simplified form, predicts universal relationships linking final eye size and developmental time to a single parameter which integrates genetically-controlled variables, the rates of cell proliferation and differentiation, with geometrical factors. We find that the predictions of the theoretical model show good agreement with previously published experimental results. We also develop a new computational model that recapitulates the process more realistically and find concordance between this model and theory as well, but only when the primordium is circular. However, when the primordium is elliptical both models show discrepancies. We explain this difference by the mechanical interactions between cells, an aspect that is not included in the theoretical model. Globally, our work defines the quantitative relationships between rates of growth and differentiation and organ primordium size that ensure growth termination (and, thereby, specify final eye size) and determine the duration of the process; identifies geometrical dependencies of both size and developmental time; and uncovers potential instabilities of the system which might constraint developmental strategies to evolve eyes of different size.

## 1. Introduction

The control of organ size and shape (i.e., the organ's morphology) in animals constitutes a fundamental process that is still not very well understood (Hafen and Stocker, 2003; Eder et al., 2017). And this is because it poses something of a conundrum: on the one hand, organ morphology is remarkably constant within a given species. Even in cases in which environmental factors affect the species-specific organ size and shape, this so-called organ plasticity usually follows definite rules. On the other, the same organ in different species may exhibit striking size differences (reviewed in Mirth et al., 2016; Vollmer et al., 2017a). Therefore, mechanisms must exist that ensure morphological constancy within a species but which allow for morphological variation during evolutionary diversification.

Although organ growth is coordinated with the overall growth of the individual, it is often the case that the size of an organ is largely controlled in an organ-autonomous manner. That is, it depends on its own genetic constitution. This is illustrated by classic experiments by Twitty and Schwind (1931) in which limb rudiments from a species of small salamander were grafted onto a developing salamander of a much larger species. The grafted limb grew just to the small size typical of the small species (Twitty and Schwind, 1931) despite its having developed in the context of a larger individual. This experiment highlights the two major questions behind organ growth control: how organs grow to a species-specific size and how organ size varies in different species. In this paper, we try to contribute to addressing these questions by studying a simple model, the eye of the vinegar fly *Drosophila melanogaster*.

**Figure 1:**
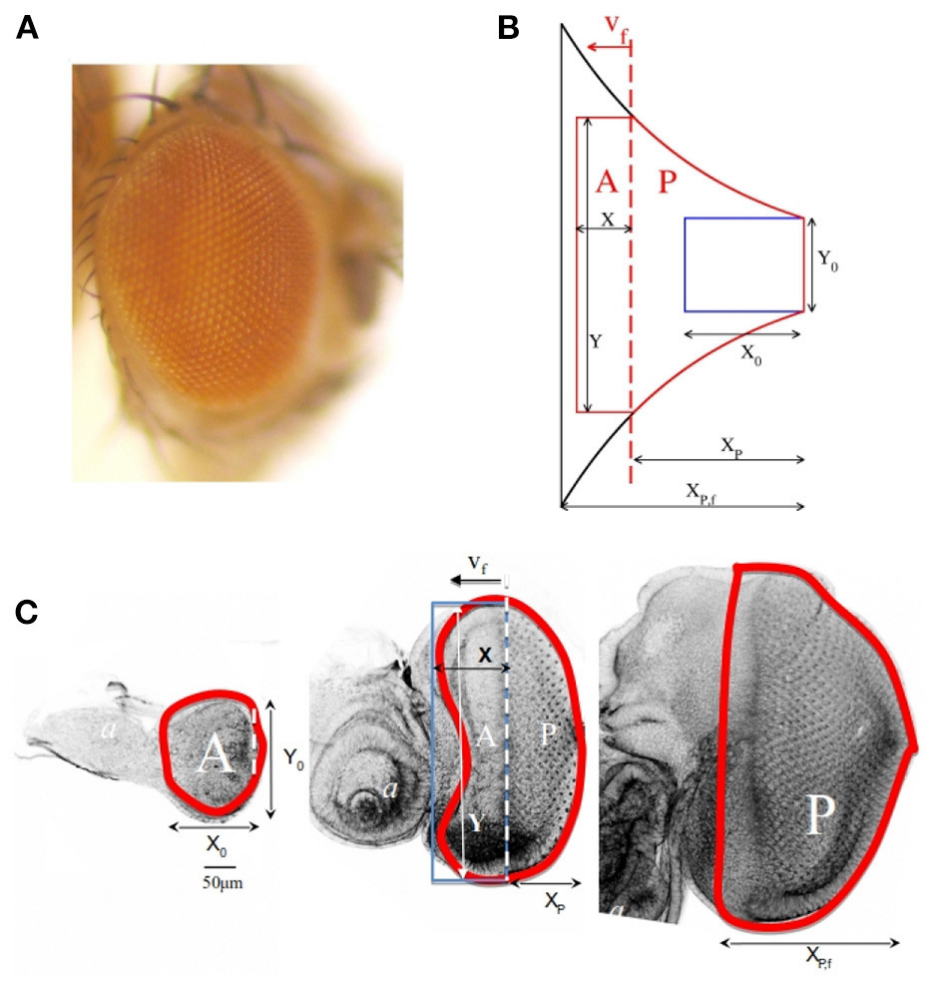
**(A)** Adult eye in *Drosophila melanogaster*. **(B)** Schematic representation of the shape of the eye according to the theoretical model. The blue rectangle corresponds to the primordium shape at *t* = 0. The black contour represents the final shape of the eye. The red curve is the shape of the eye at an intermediate stage. **(C)** Eye imaginal discs from early (left), mid (middle) and final (right) third larval stage, stained with Rhodamine-Phalloidin, to mark cell contours, and imaged using a Leica SPE confocal set up. Images were processed with Adobe Photoshop. In these figures *X*_0_ and *Y*_0_ correspond to the initial dimensions of the primordium. The morphogenetic furrow (MF) is represented as a vertical red dashed line that separates the anterior *A* from the posterior *P* region and it moves with speed *v*_*f*_. The instantaneous and final posterior-to-anterior dimensions of *P* area, *X*_*P*_ and *X*_*P,f*_, as well as the instantaneous dimension of *A*, *X*, and *Y*, are also represented in the figures.

The eye of *D. melanogaster* is of the compound type, typical of insects and crustaceans and the most common eye architecture in nature (Land and Nilsson, 2012). In compound eyes, retinal cells are arranged in stereotypical clusters, called ommatidia. Each ommatidium is a unit eye formed by a constant number of photoreceptors, pigment and lens-secreting cells, totaling 16 cells. About 800 ommatidia are packed together forming the dome-shaped eye of the *Drosophila* adult, so that its final size in cell number is about 13,000 cells. However, eye size (as well as eye shape) varies in different *D. melanogaster* strains, a variation that is more striking if the whole order of flies (Diptera) is considered, with ommatidia number ranging from a few tens to tens of thousands per eye in different fly species (Casares and McGregor, 2021). The eye in flies develops during the life of the larva from a monolayered epithelium (called eye imaginal disc). Therefore, the growth and differentiation of the eye is, essentially, a bi-dimensional process. After a period in which the primordium grows by cell division, differentiation starts as a signaling wave sweeps across the primordium from posterior to anterior (Treisman, 2013). The wave front is characterized by an indentation of the epithelium and is called “morphogenetic furrow” (MF) (Tomlinson, 1985 and Figure 1).

Interestingly, at any time during the process, the MF separates cells in two states: anterior to the MF lie the proliferative progenitor cells; posterior to it these progenitors exit the cell cycle (that is, they halt proliferation) and differentiate. This means that growth is sustained by the proliferation of cells anterior to the MF. Only when the MF reaches the anterior-most edge of the primordium and the pool of progenitor cells is exhausted the eye reaches its final size. Note that, although the final number of ommatidia depends on the number of cells produced, the size of the adult eye also depends on ommatidial size (Cagan, 2009). Also, there exist apoptotic events that eliminate excess cells once the differentiation wave has passed. However, since this apoptosis does not affect the number of ommatidia, it has little impact on final eye size (Cagan and Ready, 1989). A great deal of work has characterized the pathways controlling eye development *D. melanogaster* (henceforth *Drosophila*) (Treisman, 2013; Casares and Almudi, 2016). Although the process of eye development has not been studied *in vivo* (or *ex vivo* in culture), it can be followed by dissecting the eye primordium out of the larva at different times during its development, so that a complete dynamic picture of its development can be reconstructed. The relative simplicity of the developing fly eye, together with its experimental accessibility, make it a good model system to understand how organ morphology is controlled in epithelial-derived organs where growth depends on a balance between the rates of progenitor proliferation and differentiation.

From a biological perspective, as we mentioned above, there are several general questions regarding organ size that need to be addressed. The first one is under what conditions does an organ stop growing and attain a final, species-specific size. The second one is which biological parameters (and to what extent) need to change, in order to generate the variety of organ sizes seen in nature. The third question concerns the relationship between organ size and shape, i.e., how these elements affect each other during organ development. Recent work on the *Drosophila* eye has been aimed at answering some of these questions. Wartlick et al. (2014) and Vollmer et al. (2017b) proposed two alternative mechanisms, based on the dynamics of growth control pathways, as ways to explain the cessation of eye primordium growth and, thus, the control of final eye size. In addition, Fried and coworkers (Fried et al., 2016) modeled a simplified gene network known to control the recruitment of progenitors and the movement of the differentiation wave on an elliptic growing domain, as an approximation of the eye primordium. This model reproduced well quantitative data on growth/differentiation dynamics of the eye primordium, showing that the known genetic relationships suffice to explain the genetic control of eye size.

However, in order to be of greater use for biologists, these models should be able to, on the one hand, yield quantitative expressions relating biological rates (such as growth rate, differentiation wave speed, etc.) with which to make predictions about which biological variables control growth termination and final organ size, and which quantitative changes in these variables might underlie variation of organ size. On the other hand, such models should explore explicitly the dependency between eye size and shape.

In this paper we have attempted to tackle these issues. First, we developed a simple phenomenological model, using a minimal set of assumptions, that relates major genetically-controlled variables, such as rates of cell growth and division or differentiation speed, and the initial shape of the organ, a parameter of “geometrical” character. This latter is ultimately also under genetic control, although the link between genes and geometry is still elusive. The analysis of our model predicts universal relationships for final eye size and associated developmental time and shows good qualitative and quantitative agreement with *Drosophila* eye growth/differentiation dynamics previously obtained experimentally (Vollmer et al., 2016), despite the reduced number of aspects considered. The simple quantitative relationships obtained with this model can be used as a guide to predict experimental values of the relevant parameters when the aim is to study the causes of eye size variation. However, this model is too rigid and the relationship between size and shape cannot be addressed. Additionally, we developed a computational individual-based model (ibM) to simulate the growth and differentiation dynamics of the primordium taking into account its shape.

## 2. Materials and Methods

### 2.1. Experimental Methods

The *Drosophila melanogaster* wild type strain *Oregon-R*, the *GMR-GAL4* and *GMR-GAL4;UAS-Upd* genotypes are as in Vollmer et al. (2016). Cultures were maintained at 25°C in standard fly media. Heads from 2 to 3 days old adult flies (males and females) were dissected and mounted in Hoyer's medium: Lactic Acid (1:1) as in Wieschaus and Nusslein-Volhard (1999). Frontal and occipital planes were imaged under a Leica DM5000 microscope with a 20X objective using a Leica 490 digital camera. Digital images were stored as .tif files. The frontal and occipital areas of the left and right eyes, as well as the total head area were measured for each head using the polygonal tool in Fiji (Schindelin et al., 2012) and expressed in arbitrary units. Total eye area results from adding the frontal and occipital eye areas.

Statistical was carried out using the R software framework (version 4.0.1). We analyzed a normalized eye area, by using the total head area (*H*) as a correlate of body size. The eye/head ratio is then defined as:

(1) $$\begin{array}{l} EHr=\frac{R+L}{2H} \end{array}$$

with *R* and *L* the area of right and left eyes. *EHr* groups comparison was made using the ANOVA Welch corrected method in order to deal with heteroscedasticity across groups and implemented by *oneway.tes*t R internal function. Pairwise comparisons among groups were performed with Welch's test with Holm's correction, implemented by *pairswise.t.test* R internal function (*pool.sd* = *F, p.adjust.method* = “*Holm”*). Variance comparison was performed by Levene test. *LeveneTest* from R package car.

### 2.2. Simulation Methods

As an alternative to the theoretical model, and to gain more insight into the eye growth process, we have developed an IbM model to attempt to realistically simulate *Drosophila* eye growth. For our computational model we have adopted an ibM model, similar in concept to the proposed for some of us to study the evolution of bacterial biofilms (Acemel et al., 2018), with some relevant modifications. In our case, cells have been modeled as the bidimensional projections of very small spherocylinders of cylinder elongation *L* and hemispherical ends of σ diameter (see Results section). In this model cells interact with each other following a Kihara potential (Kihara, 1963). This interaction potential, used in the past for the simulation of elongated colloidal particles (Vega and Lago, 1990; Cuetos et al., 2003), is repulsive at short distances, reproducing steric repulsion between cells at contact, and attractive when the distance between the particles is not very long to resemble intercellular adhesion. In our model, this interaction potential was truncated and shifted at distance of 3σ. The width of the attractive well of the potential was set to 10*k*_*B*_*T*. Cell movement is also influenced by thermal agitation, which is simulated using Brownian Dynamics (BD) (Löwen, 1994). In BD simulations, the particle trajectories are obtained by integrating the Langevin equation forward in time. The trajectories of the center of mass of an individual cell **r**, and the orientation of its longitudinal axis **û**, evolve in time according to the following set of equations:

(2) $$\begin{array}{l} \begin{array}{l} {\text{r}}^{\left|\right|}\left(t+\Delta t\right)={\text{r}}^{\left|\right|}\left(t\right)+\frac{D_{\left|\right|}}{k_{B}T}{\text{F}}^{\left|\right|}\left(t\right)\Delta t+\\ \;+{\left(2D_{\left|\right|}\Delta t\right)}^{1/2}R^{\left|\right|}\hat{\text{u}}\left(t\right) \end{array} \end{array}$$

(3) $$\begin{array}{l} r^{⊥}(t+\Delta t)=r^{⊥}(t)+\frac{D_{⊥}}{k_{B}T}F^{⊥}(t)\Delta t+\\ \;+{\left(2D_{⊥}\Delta t\right)}^{1/2}(R_{1}^{⊥}{\hat{\text{v}}}_{1}(t)+R_{2}^{⊥}{\hat{\text{v}}}_{2}(t)) \end{array}$$

(4) $$\begin{array}{l} \hat{\text{u}}(t+\Delta t)=\hat{\text{u}}(t)+\frac{D_{\vartheta }}{k_{B}T}T_{b}(t)\times \hat{\text{u}}(t)\Delta t+\\ \;+{\left(2D_{\vartheta }\Delta t\right)}^{1/2}(R_{1}^{\vartheta }{\hat{\text{w}}}_{1}(t)+R_{2}^{\vartheta }{\hat{\text{w}}}_{2}(t)) \end{array}$$

being **r**^∥^ and **r**^⊥^ the projections of **r** on the directions parallel and perpendicular to **û**, respectively. **F**^∥^ and **F**^⊥^ are the parallel and perpendicular components of the total force acting on *b* and **T** is the total torque due to the interactions with other particles of the fluid (Vega and Lago, 1990). The particle (cell) Brownian dynamics is induced through a set of independent gaussian random numbers of variance 1 and zero mean: *R*^∥^, $R_{1}^{⊥}$, $R_{2}^{⊥}$, $R_{1}^{\vartheta }$ and $R_{2}^{\vartheta }$, and unitary vectors perpendicular to **û**, denoted above as **v^**_*m*_ and **ŵ**_*m*_ (*m* = 1, 2). The diffusion coefficients, *D*_∥_, *D*_⊥_ and *D*_ϑ_ were calculated employing the analytical expressions proposed by Shimizu for prolate spheroids (Shimizu, 1962; Acemel et al., 2018). These depend on a diffusion parameter $D_{0}=D_{0}^{*}\sigma^{2}/\tau$, setting $D_{0}^{*}=0.1$. According to Acemel et al. (2018), this value favors the formation of compact cells clusters. In all the simulations the time step was fixed as Δ*t* = 10^−6^τ. τ is the unit of time.

As initial configuration for the simulations we have used mainly a circular ensemble of 200 particles (“cells”) of length *L*_0_, although we have also carried out simulations with ellipsoidal primordia with prolate (major axis parallel to MF) or oblate shape (major axis perpendicular to MF). To generate these initial configurations we have carried out a simulation with an algorithm similar to the one described above, but with synchronous elongation of the particles and no morphogenetic furrow. With this procedure, a cluster of 4,000 particles with *L*_0_ was generated. From this cluster, a region with the desired geometry and number of cells was selected as initial configuration. Examples of snapshots of initial configurations are shown in **Figures 3, 6**. For each case, numerical values have been averaged over 10 independent simulations with the same initial configuration and parameters but different random number seeds.

## 3. Results

### 3.1. Theoretical Model

Here we present a simple mathematical model of *Drosophila* eye growth and differentiation. The general growth characteristics of the *Drosophila* eye have been described in the introduction. Before delving into our theoretical approach, it is convenient to highlight some of the characteristics of this process and recall some of the basic facts.

Firstly, the larval eye primordium is assumed to be a flat two-dimensional surface. This approach is justified because the eye primordium is an epithelial monolayer. Previous quantitative data support that this analogy is valid (Vollmer et al., 2016). Another fundamental aspect is the movement of a differentiation wave, also known as the morphogenetic furrow (MF), that starts at the posterior pole of the primordium and sweeps toward anterior. The time of differentiation onset is set as *t* = 0. Movement of the MF divides the eye primordium (*T*) into two areas: one ahead of the differentiation MF (*A*), where undifferentiated cells proliferate, other behind it (*P*), where cells that have been overtaken by the MF cease proliferation. This sequence, indicating the defined regions along the growth of the embryonic eye, as well the final result of and adult eye, are shown in Figure 1C.

Based on these ideas, in our theoretical model the primordium at the time of differentiation onset is represented by a rectangle with initial dimensions *X*_0_ and *Y*_0_ for the anterior-posterior and dorsal-ventral axes, respectively. We have considered the MF as a line perpendicular to the anterior-posterior axis that moves from posterior to anterior at constant speed *v*_*f*_. The MF line divides the total area (*T*) into two subdomains: an anterior area (*A*) and a posterior area (*P*). The first one expands due to cell proliferation (we assume isotropic growth characterized by a time-independent constant rate *k*) and diminishes as a result of MF advance. The posterior area, on the contrary, increases in size as a result of MF movement. Along this process, the *A* region keeps a rectangular shape, with dimensions *X* and *Y* that depend on time *t*. This means that at each instant *A* = *X* · *Y*. *Y* is in turn the instantaneous length of the MF. The shape of *P* will be an output of the model. In Figure 1B the main features of our model are sketched. Figure 1C indicates the equivalence of the dimensions defined in the model with those of a growing eye primordium. From this description, it follows that

(5) $$\begin{array}{l} T=A+P\to \frac{dT}{dt}=\frac{dA}{dt}+\frac{dP}{dt} \end{array}$$

where the time derivative of *P*, due to the movement of the MF corresponds to

(6) $$\begin{array}{l} \frac{dP}{dt}=v_{f}\cdot Y \end{array}$$

while for *A*, in addition to the MF movement, it is necessary to introduce the influence of cell proliferation

(7) $$\begin{array}{l} \frac{dA}{dt}=k\cdot A-v_{f}\cdot Y \end{array}$$

When the definition of *A* is introduced in the previous equation

(8) $$\begin{array}{l} \frac{d\left(X\cdot Y\right)}{dt}=k\cdot \left(X\cdot Y\right)-v_{f}\cdot Y \end{array}$$

by separation of variables, and taking into account isotropy in the cell proliferation, two equations for *X* and *Y* are obtained

(9) $$\begin{array}{l} \frac{dX}{dt}=\frac{k\cdot X}{2}-v_{f};\frac{dY}{dt}=\frac{k\cdot Y}{2} \end{array}$$

Integrating these equations from the initial values *X*_0_, *Y*_0_ at *t* = 0 it is easy to arrive to the following expressions

(10) $$\begin{array}{l} X=\left(X_{0}-2\frac{v_{f}}{k}\right)e^{\frac{k\cdot t}{2}}+2\frac{v_{f}}{k}=\left(X_{0}-2F\right)e^{\frac{k\cdot t}{2}}+2F \end{array}$$

(11) $$\begin{array}{l} Y=Y_{0}\cdot e^{\frac{k\cdot t}{2}} \end{array}$$

where a new variable *F* = *v*_*f*_/*k* has been defined. Hence

(12) $$\begin{array}{l} A=XY=Y_{0}\left(X_{0}-2F\right)e^{k\cdot t}+2Y_{0}Fe^{\frac{k\cdot t}{2}} \end{array}$$

Introducing Equations (6, 7) in Equation (5) we can write *dT*/*dt* = *kA*. Then, the dependence of *T* with *t* is obtained integrating from the initial value *T*_0_ = *A*_0_ = *X*_0_ · *Y*_0_

(13) $$\begin{array}{l} T=T_{0}+Y_{0}\left(X_{0}-2F\right)\left(e^{k\cdot t}-1\right)+4Y_{0}F\left(e^{\frac{k\cdot t}{2}}-1\right) \end{array}$$

If now we define the new variable $\tilde{F}=F/X_{0}$, the time dependency of *T* and *A* can be expressed in a more concise way

(14) $$\begin{array}{l} \begin{array}{l} A=T_{0}\left(\left(1-2\tilde{F}\right)e^{k\cdot t}+2\tilde{F}e^{\frac{k\cdot t}{2}}\right)\\ \;=T_{0}\left(\left(1-2\tilde{F}\right)e^{\frac{X_{P}}{F}}+2\tilde{F}e^{\frac{X_{P}}{2F}}\right) \end{array} \end{array}$$

and

(15) $$\begin{array}{l} \begin{array}{l} T=T_{0}\left(1+\left(1-2\tilde{F}\right)\left(e^{k\cdot t}-1\right)+4\tilde{F}\left(e^{\frac{k\cdot t}{2}}-1\right)\right)\\ \;=T_{0}\left(1+\left(1-2\tilde{F}\right)\left(e^{\frac{X_{P}}{F}}-1\right)+4\tilde{F}\left(e^{\frac{X_{P}}{2F}}-1\right)\right) \end{array} \end{array}$$

Here we have denoted the instantaneous posterior length as *X*_*P*_. As *X*_*P*_ = *v*_*f*_ · *t* it is immediate to obtain that $k\cdot t=F^{-1}\cdot X_{P}$. This change of variable is convenient for the comparison with the experimental results, which are usually expressed as a function of *X*_*P*_ rather than *t* (Vollmer et al., 2016, 2017b), as the more developmental time elapses, the longer *X*_*p*_ will be. This means that the area growth rate reported in Vollmer et al. (2016) is related with our theoretical model through the expression *k*′ = *F*^−1^.

We are now ready to extract some predictions from our model. The first one is the necessary condition for eye growth termination, that is, for *A* = 0. This implies that the MF reaches the anterior edge of the primordium. As in Equation (14) the second term in *A* is defined as positive, it is necessary that $1-2\tilde{F}<0$ so that at some instant *A* has null value. As a consequence, eye growth completion is reached when *v*_*f*_ is larger than a limit value *v*_*f,l*_ defined as

(16) $$\begin{array}{l} v_{f}>v_{f,l}=\frac{k\cdot X_{0}}{2} \end{array}$$

If this condition is fulfilled, the time required to complete eye growth (*t*_*f*_) is that at which *A* = 0. From Equation (14)

(17) $$\begin{array}{l} k\cdot t_{f}=F^{-1}\cdot X_{P,f}=2\cdot ln\left[\frac{2\tilde{F}}{2\tilde{F}-1}\right] \end{array}$$

which is only dependent on $\tilde{F}$. *X*_*P,f*_ is the final width of the eye. On the other hand, by substituting this final time *t*_*f*_ in Equation (15), the relative final area of the full eye, *T*_*f*_/*T*_0_, is

(18) $$\begin{array}{l} \frac{T_{f}}{T_{0}}=\frac{2\tilde{F}}{2\tilde{F}-1} \end{array}$$

that is also a universal function on $\tilde{F}$.

As indicated above, the model assigns a rectangular shape to the *A* region. *P* presents an exponential contour. Combining all the results, the equations for the shape of the eye resulting from the model are

(19) $$r_{y}=\{\begin{array}{ll} 0.5\cdot Y_{0}\cdot e^{\frac{k\cdot r_{x}}{2\cdot v_{f}}} & \text{if }r_{x}<v_{f}\cdot t\\ 0.5\cdot Y_{0}\cdot e^{\frac{k\cdot t}{2}} & \text{if }r_{x}\geq v_{f}\cdot t \end{array}$$

*r*_*x*_ and *r*_*y*_ are the cartesian coordinates of the points in the contour of the eye. In Figure 1B the representation of these equations at different times is shown.

**Figure 2:**
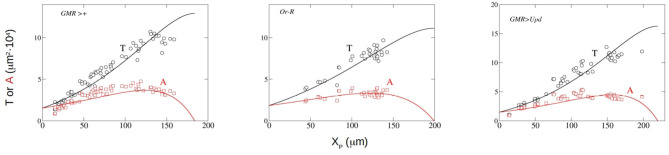
Experimental measurement of *P* (black circles) and *A* (red squares) along with least-square fitting results to Equations (15) (black line) and (14) red line for the *Drosophila* strains *GMR*>+*, Or-R* and *GMR*>*Upd*. In the three panels, the x-axis represents *X*_*P*_ in μ*m* and the y-axis depicts *A* and *P* in 10^−4^ · μ*m*^2^.

Now we can compare the predictions of our theoretical model with the experimental evidence. We have adjusted the experimental results for three strains of *Drosphila melanogaster*. The three strains analyzed are *Oregon-R* (*Or-R*), *GMR-GAL4* (*GMR*>+) and *GMR-GAL4;UAS-Upd* (*GMR*>*Upd*). These strains were used and described in Vollmer et al. (2017b). The quantitative data used were those reported in the paper. Basically, *Or-R* is a wild type strain, while *GMR-GAL4* is a control strain. In the *GMR-GAL4;UAS-Upd* strain the mitogen Upd is expressed in the developing eye which results in eye overgrowth (see Vollmer et al., 2017b). We have fitted the published results of the dependence of *A* and *P* on the anterior-posterior dimension of *P*, *X*_*P*_, to Equations (14, 15), respectively. We have employed a nonlinear least-squares method, using the function lsqnonlin from MATLAB (2020). In Table 1 the values of the parameters that are directly obtained from the adjustment to the experimental results ($T\left(0\right),\tilde{F}$ and *F*) are listed. We also include in this table the values of other magnitudes that are calculated using expressions derived in section 3.1, such as *X*_0_, *X*_*P,f*_ and *T*_*f*_. The correlation coefficient *r*^2^, that indicates the goodness of the adjustment, is also shown. The experimental data for *P* and *A* and the theoretical curves with the parameters obtained from the fitting are compared in Figure 2.

**Table 1:** Results of the adjustment of the geometric model presented in this article to the experimental results on the *Drosophila melanogaster* strains *Oregon-R* (*Or-R*), *GMR-GAL4* (*GMR*>+) and *GMR-GAL4;UAS-Upd* (*GMR*>*Upd*) from Vollmer et al. (2017b).

	***T*_0_**	**$\tilde{F}$**	**F**	***r*^2^**	***X*_0_**	***X*_*P,f*_**	***T*_*f*_**
*Or-R*	18, 378	0.599	55.249	0.801	92.235	199.19	111, 478
*GMR>+*	15, 321	0.567	43.103	0.898	76.020	183.88	129, 316
*GMR>Upd*	14, 808	0.550	45.872	0.854	83.403	220.01	162, 888

*T_0_ and T_f_ are expressed in μm^2^; F, X_0_ and X_P,f_ in μm. $\tilde{F}$ is dimensionless. r^2^ is the correlation coefficient*.

From the visual inspection of Figure 2, the theoretical model reproduces the kinetics of both *T* and *A* in the three strains for which experimental data are available, and shows that the functional dependence of *T* and *A* with *X*_*P*_ is well described by our model. This good fitting (Table 1), that includes good predictions of *T*_0_ and *T*_*f*_, is achieved using a constant growth rate *k*, in contrast with previous models that used non-constant, decaying growth rates (Vollmer et al., 2016). Further experimentation is needed to clarify this issue.

Globally, the mathematical model, which shows good fit to experimental data, predicts that: (1) eye differentiation terminates only if the ratio *v*_*f*_/*k* is larger than half the width of the primordium (*X*_0_), which highlights the importance that the shape of the primordium has on the final organ's size; (2) the relative increase in size of the eye during its development (*T*_*f*_/*T*_0_) as well as the time required for eye development (from *T*_0_ to *T*_*f*_), *t*_*f*_, are both determined by *v*_*f*_, *k* and *X*_0_, with larger *T*_*f*_/*T*_0_ requiring longer *t*_*f*_; (3) finally, and as will become more obvious later on when we discuss a computational model of the process, the impact of variations in *v*_*f*_, *k* and *X*_0_ on *T*_*f*_/*T*_0_ and *t*_*f*_ are non linear, which suggests that eyes of different size should be differentially sensitive to developmental noise. However, while the model predicts well the developmental trajectory of eye growth, it fails in predicting eye shape accurately: the modeled eye is trapezoidal, while insect eyes are approximately ellipsoidal. To be able to address directly questions related to shape, we decided to adopt a more flexible modeling approach, using individual-based computational model (IbM).

### 3.2. Individual Based Model to Explicitly Explore the Relationship Between Size and Shape

To extend our modeling beyond the rigidity of our theoretical model, we built an IbM model in which the cells in the developing eye are modeled as the bidimensional projection of very short spherocylinders. An spherocylinder is a cylinder of elongation *L* with hemispherical ends of diameter σ. This is a widely used model in computer simulation of liquid crystals (Allen et al., 1993; Cuetos and Martínez-Haya, 2015), and it has been previously applied to model bacterial biofilms (Acemel et al., 2018). Consequently, the total length of the cell is *L* + σ. In this algorithm the particles are quasi-circular to model epithelial cells more accurately, with initial length $L_{0}=10^{-9}\sigma +\sigma$. Those cells that have not been reached by the MF grow in length at constant velocity. This velocity $v_{gr}^{i}$ for each cell is selected at random at the moment of the division from a gaussian distribution with mean *v*_*gr*_ and relative standard deviation *s*/*v*_*gr*_ = 0.1. When a cell reaches a length *L*_*f*_ = 2 · *L*_0_, it divides into two new cells with the original length *L*_0_. If these daughter cells have not been surpassed by the MF, they continue this cycle of elongation and division. The growth direction of the daughter cells does not correlate with that of the mother cell, being randomly selected at the time of the division. The MF is an imaginary vertical line that moves from the posterior to the anterior edge of the ensemble of cells at constant velocity *v*_*f*_. Cells that are surpassed by the MF finish their cycle of growth and division, producing final daughter cells that do not longer grow or divide, maintaining their original length *L*_0_. First, we are going to compare the theoretical predictions with simulation results in the case of a circular primordium. Next we will investigate the effect of the shape of the primordium on those variables.

**Figure 3:**
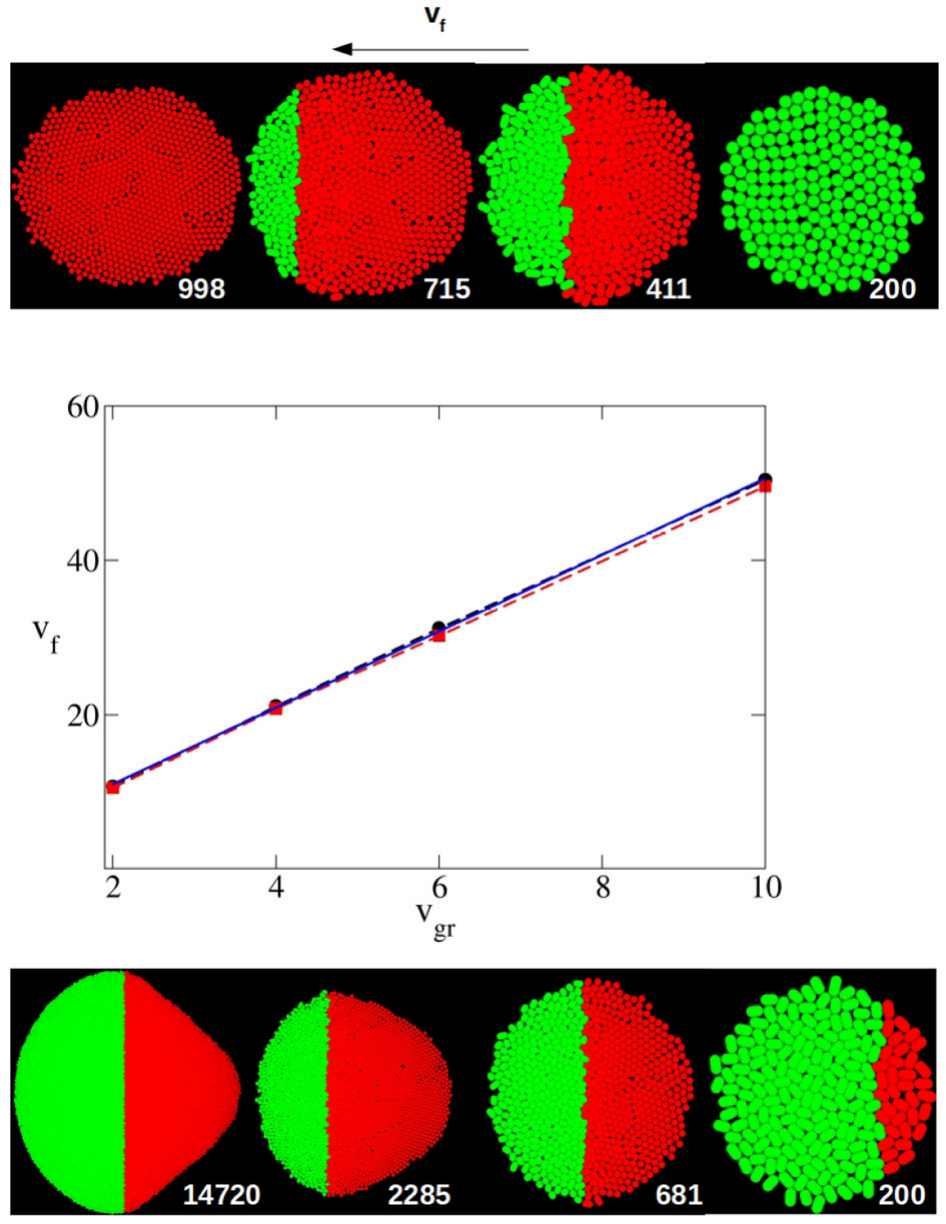
Middle panel: Dependence of the highest and lowest value of *v*_*f*_ for which eye growth termination does not occur ($v_{f}^{nc}$, red square and dashed line) or does take place ($v_{f}^{c}$, black circles and dashed line) with *v*_*gr*_ as obtained by computer simulation for a circular primordium. The solid blue line is the limiting theoretical value of *v*_*f*_, *v*_*f,l*_, from Equation (16). Both *v*_*f*_ and *v*_*gr*_ are expressed in units of σ/τ. Top and bottom panel: From right to left, sequence in the evolution of the eye as obtained with the simulation algorithm. In the top panel an eye that terminates its growth (*v*_*gr*_ = 6σ/τ, *v*_*f*_ = 37σ/τ, *X*_0_ = 14.7σ and $\tilde{F}=0.606$). In the bottom panel the development of an eye that does not terminate growth (*v*_*gr*_ = 6σ/τ, *v*_*f*_ = 25σ/τ, *X*_0_ = 14.7σ and $\tilde{F}=0.409$). In both panels, the number of particles (cells) is printed close to each snapshot. Both cases start with the same circular primordium (top right snapshot).

For instance, Equation (16) indicates a condition that *v*_*f*_ must meet for eye growth termination. If this condition is not satisfied, the MF will not reach the anterior pole and the eye primordium will continue to grow indefinitely. Does the computational model reproduce this condition? And if it does, what is the degree of agreement between the two models? In the central panel of Figure 3, for various values of *v*_*gr*_ and in the case of a circular primordium, the highest value of *v*_*f*_ for which, in computer simulation experiments, eye growth does not finish, $v_{f}^{nc}$, is represented. The lowest value of *v*_*f*_ for which the end point is reached, $v_{f}^{c}$ is also represented. $v_{f}^{nc}$ and $v_{f}^{c}$ correspond, respectively, to the upper and lower values of *v*_*f*_ for which all the replicates reach (or not) the termination of eye growth. In this figure, the limiting value *v*_*f,l*_ as a function of *v*_*gr*_ (Equation 16) is also plotted. To compare theory with simulations we need to determine the values of *k* and *X*_0_ from the computer simulation input parameters. As we have postulated that the cell division shows a first order kinetics, and considering that the time required to double the population of cells in the simulation run can be approximated to the average time that an individual cell takes to complete the growth and division cycle, it is easy to obtain a relation between the mean value of the growth velocity *v*_*gr*_ and the kinetic constant *k*:

(20) $$\begin{array}{l} k=\frac{ln\left(2\right)\cdot v_{gr}}{\sigma } \end{array}$$

In computer simulation experiments *X*_0_ is computed as the maximal distance between the center of two cells at the opposite poles of the initial primordium on the anterior-posterior axis. From the initial configuration used in our simulations in the case of a circular primordium, this value is *X*_0_ = 14.7σ. With these considerations, in Figure 3 it can be observed that the theoretical prediction of *v*_*f,l*_ is in very good agreement with the results obtained by computer simulation in these cases. Hence, the values of $v_{f}^{nc}$ and $v_{f}^{c}$ obtained by simulation are very close. And the theoretical value of *v*_*f,l*_ is almost coincident in a broad range of *v*_*gr*_ values. In both theory and simulation, the linear dependence between the limiting value of *v*_*f*_ and *v*_*gr*_ is clearly observed.

Figure 3 also shows a sequence of snapshots that illustrates the development of the system in a case where the termination is reached (top panel) and another where the eye grows without end (bottom panel). In these sequences it can be observed how the front of the *P* region (red particles) moves as if it were the MF (right to left in these figures). While in the sequence in the top panel the MF reaches the anterior pole and cell proliferation finishes, in the case of the sequence shown in the bottom panel the MF never reaches the anterior pole, and, as a consequence, the *A* region still keeps proliferating.

**Figure 4:**
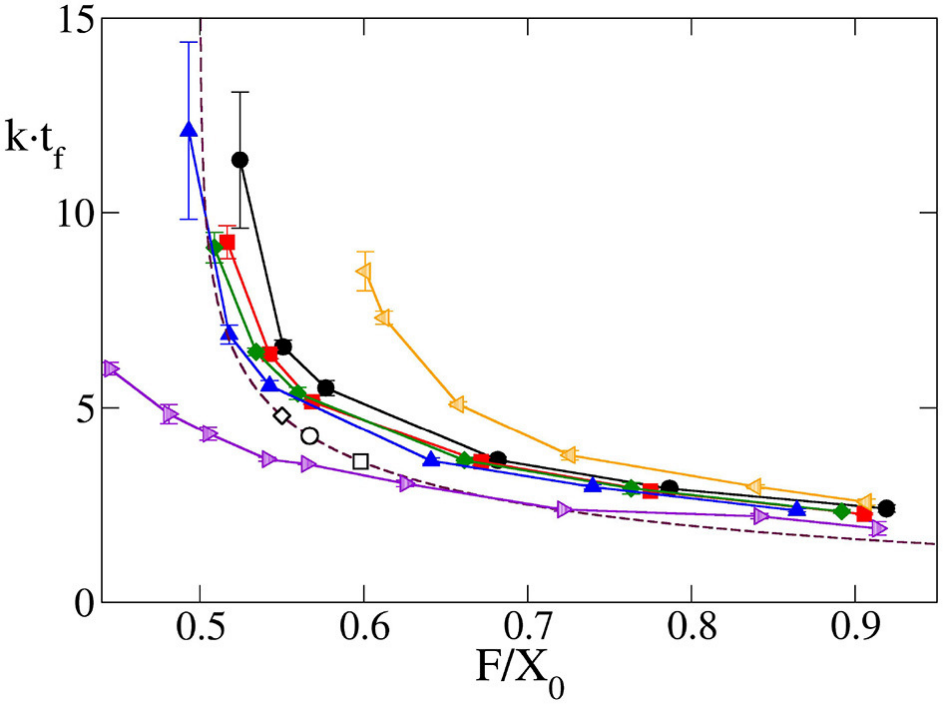
Time required to complete eye growth multiplied by the constant growth rate (*k* · *t*_*f*_), as a function of $\tilde{F}=F/X_{0}$ obtained by computer simulation for the case of circular primordium and *v*_*gr*_ = 2σ/τ (black line and circles), 4σ/τ (red line and squares), 6σ/τ (green line and diamonds) and 10σ/τ (blue line and triangles). Violet right triangles correspond to oblate primordium with *X*_0_/*Y*_0_ = 0.540, orange left triangles correspond to prolate primordium with *X*_0_/*Y*_0_ = 1.923. In these last two cases *v*_*gr*_ = 6σ/τ. Symbols are simulation results, and solid lines are guidance for the eyes. Additionally, the universal law Equation (17) is plotted as a maroon dashed line. The values of this final time for four *Drosphila* strains calculated using the parameters of Table 1 in Equation (17) are included as open circles for *GMR*>+, open squares for *Or-R* and open diamonds for *GMR*>*Upd*. Here, as well as in Figure 5, it can be seen how the results of the simulation model with circular primordium agree with the predictions of the theoretical model, while there are systematic discrepancies for the cases of non-spherical primordium. Note that as $\tilde{F}$ decreases the finalization time *t*_*f*_ increase, and this increase is non-linear.

Another interesting output variable is developmental time, *t*_*f*_, as the time elapsed from the initiation of differentiation to full differentiation and growth termination of the eye, for the cases for which *v*_*f*_ > *v*_*f,l*_. This is highly relevant biologically, as the development of the organ should match the overall developmental time of the organism. According to Equation (17), the theoretical model predicts a universal relation between this time, multiplied by *k*, and $\tilde{F}$. In Figure 4 it can be observed that, indeed, for a wide range of *v*_*gr*_ and $\tilde{F}$, the results obtained by computer simulation for a circular primordium collapse on a universal behavior, very close to that predicted by Equation (17). There are small numeric differences between theory and simulation, especially at high values of $\tilde{F}$. These might arise from the fact that while in the theoretical model growth stops immediately behind the MF, in the computational model cells complete their last division cycle behind the MF before halting growth. This results in an additional increment of the eye's area in the latter model, which could be the reason of the observed small discrepancy. The comparison between theory and simulation shown in Figure 4 indicates that, at least for a for circular primordium, the relationship between *k* · *t*_*f*_ and $\tilde{F}$ predicted in Equation (17) holds true.

**Figure 5:**
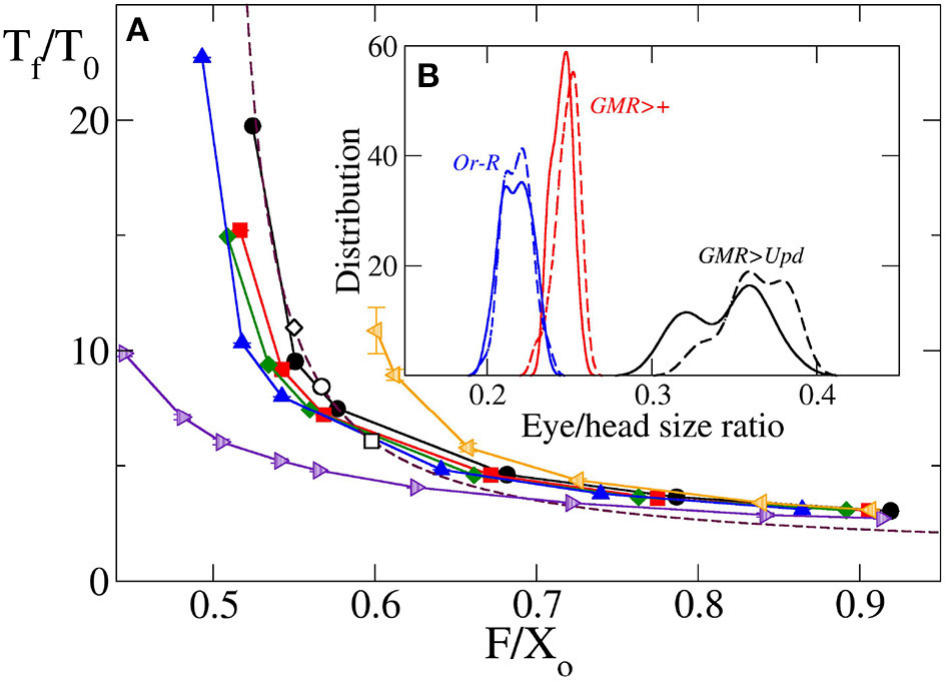
**(A)** Relative final size of the eye *T*_*f*_/*T*_0_ as a function of $\tilde{F}=F/X_{0}$ obtained by computer simulation for *v*_*gr*_ = 2σ/τ (black line and circles), 4σ/τ (red line and squares), 6σ/τ (green line and diamonds) and 10σ/τ (blue line and triangles). Violet right triangles correspond to oblate primordium with *X*_0_/*Y*_0_ = 0.540, orange left triangles correspond to prolate primordium with *X*_0_/*Y*_0_ = 1.923, in both cases with *v*_*gr*_ = 6σ/τ. Symbols are simulation results, and solid lines are guidance for the eyes. Additionally, the universal law Equation (18) is plotted as a maroon dashed line. The values of this relative final size for four *Drospila* strains calculated using the parameters of Table 1 in Equation (17) are included as open circles for *GMR*>+, open squares for *Or-R* and open diamond for *GMR*>*Upd*. Note that as $\tilde{F}$ decreases the relative final eye size increases, and this increase is non-linear. **(B)** In the inset are plotted measured values of eye head ratio distribution for *GMR*>*Upd* (black lines), *GMR*>+ (red lines) and *Or-R* strains. Solid and dashed lines correspond, respectively, to females and males. The relative standard deviation of these distributions are 0.9, 0.7, and 2.3 for *Or-R, GMR*>+ and *GMR*>*Upd* strains, respectively, without significant differences between males and females.

A similar agreement between simulation and theoretical prediction is found for the dependence of the ratio between final and initial eye size, *T*_*f*_/*T*_0_, with the parameter $\tilde{F}$. Figure 5A shows that for a circular primordium, although there are some quantitative differences, the simulation results collapse on a curve very similar to that predicted by Equation (18). The simulation estimates of *T*_*f*_/*T*_0_ were obtained considering it as equivalent to the relation between the final and the initial number of cells. In both Figures 4, 5A we have included the values obtained from the fitting to the experimental values. In any case, it is interesting to note that both *k* · *t*_*f*_ and *T*_*f*_/*T*_0_ have an asymptotic behavior for small values of $\tilde{F}$, with a large increase as $\tilde{F}$ decreases for values close to 0.5. As $\tilde{F}=F/X_{0}$ it means that, in this range of values, a small change in primordium size at the onset of differentiation (*X*_0_) would have a large impact on eye size and developmental time to termination. This result suggests that, in order to ensure robustness in organ size and developmental time, evolutionary pressure would have selected relationships between *v*_*f*_ and *v*_*g*_ away from these values. In the data set used here, the wild type/control strains *Or-R* and *GMR*>+ have values away from this sensitive region. However, *GMR*>*Upd* lies on the sensitive part of the curve. Therefore, the prediction of our model is that this strain should be more sensitive to noise, both developmental and environmental. As a consequence, it would be expected that this strain showed the highest variability in eye size. To test this, we took careful eye measurements on a new set of *Or-R, GMR*>+ and *GMR*>*Upd* flies (see Methods). Indeed, the normalized eye size (eye to head ratio) of *GMR*>*Upd* is not only larger than in the other two strains, but the variance of the distribution is also significantly larger (Figure 5B), which supports our conclusions.

**Figure 6:**
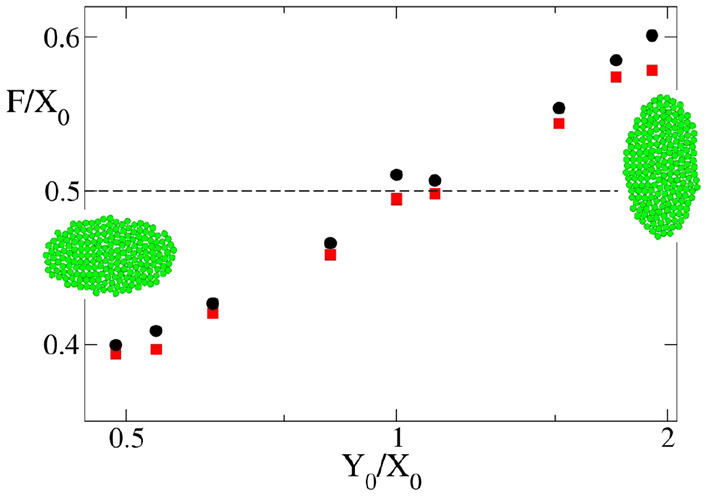
Dependence of the highest and lowest value of $\tilde{F}=F/X_{0}$ for which eye growth termination does not occur (red square) or does take place (black circles) with the shape of the primordium indicated as *Y*_0_/*X*_0_ as obtained by computer simulation. In all the cases *v*_*gr*_ = 6σ/τ. The snapshots are typical examples of primordia with *X*_0_ > *Y*_0_ and *X*_0_ > *Y*_0_. An example of the case *X*_0_ = *Y*_0_ is shown in Figure 3. The dashed line is the theoretical limiting value $\tilde{F}=F/X_{0}=0.5$.

Up to now we have shown that the theoretical and computational models agree in their predictions of final size and time to growth termination when the starting primordium used in the computational simulations is circular. However, at least in *Drosophila*, the eye primordium has been shown to be elliptic at the onset of differentiation (see Vollmer et al., 2017b and references therein). Therefore, we needed to consider non-circular primordia -i.e., *X*_0_/*Y*_0_ ≠ 1. When we simulate the development of elliptical primordia, the values of *k* · *t*_*f*_ and *T*_*f*_/*T*_0_ as a function of $\tilde{F}$ deviate from those predicted by the theoretical model. For instance, Figure 6 shows the dependence on *Y*_0_/*X*_0_ of the highest and lowest value of $\tilde{F}$ for which the development of the eye does not end and ends, respectively. Here it is possible to observe how there is a systematic divergence between the simulation results and the theoretical value of $\tilde{F}=F/X_{0}=0.5$ as soon as *X*_0_/*Y*_0_ ≠ 1. These discrepancies are also observed in Figures 4, 5A. In both figures it can be seen for both oblate (*Y*_0_/*X*_0_ < 1) and prolate (*Y*_0_/*X*_0_ > 1) initial primordia, that the dependence of *k* · *t*_*f*_ and *T*/*T*_0_ on $\tilde{F}$ deviates from that predicted by Equations (17, 18). This discrepancy is more pronounced for small values of $\tilde{F}$, tending to disappear as $\tilde{F}$ grows. This result implies that, for oblate primordia, the *v*_*f*_ can move more slowly than in a circular primordium of equal *X*_0_ and still complete differentiation. The converse is also true for prolate initial primordia. Here, faster *v*_*f*_ are needed to finalize growth.

**Figure 7:**
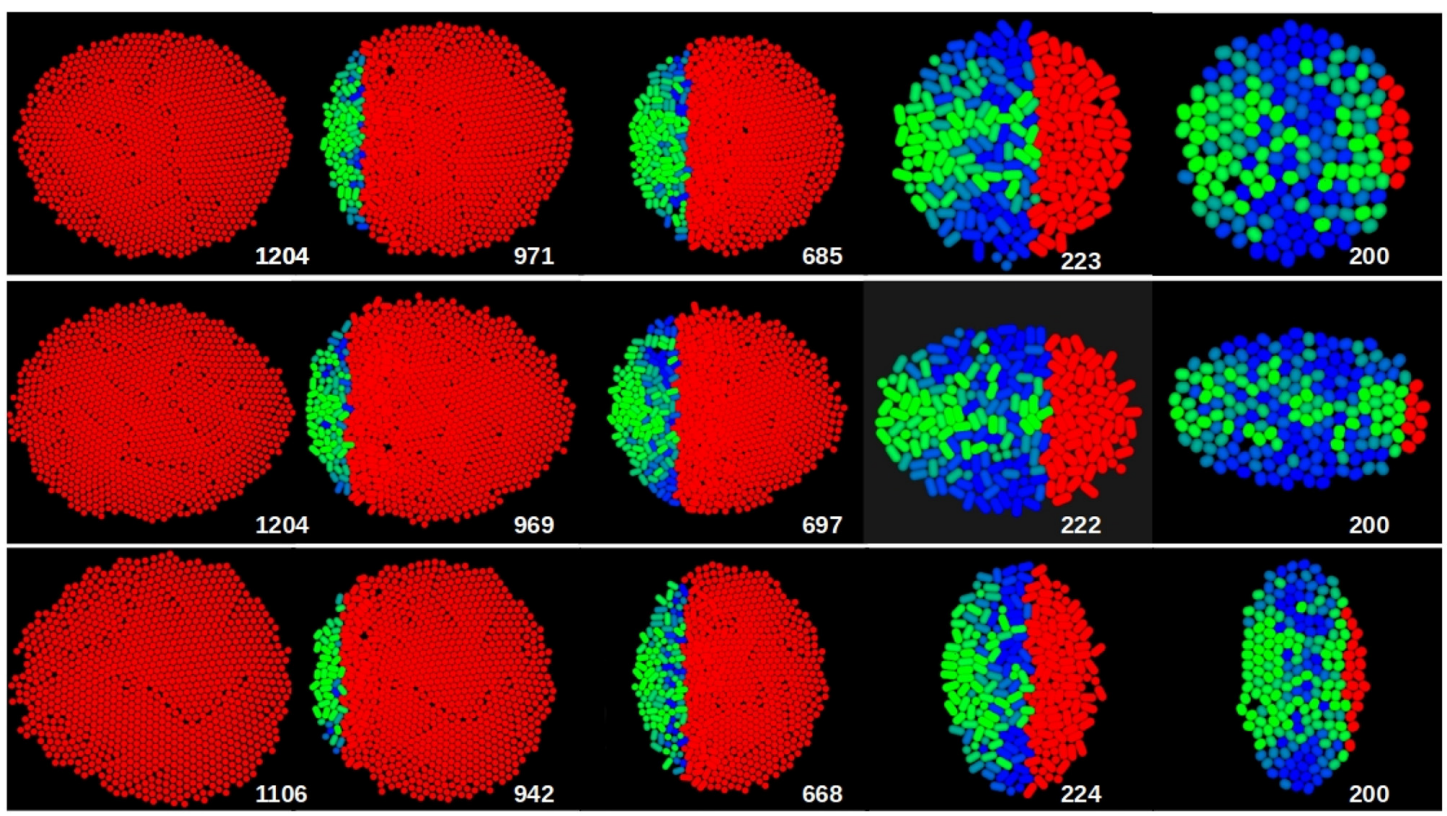
Typical sequence śof snapshots of the growth of the eye obtained by computer simulation in the case of the circular (top row), oblate (middle row) and prolate (bottom row) primordium. The number of cells is indicated in each case. The cells in *P* region are colored red. The color of the cells in *A* region is a combination between blue and green, being the green contribution the square of the component of the force over the cell, while the blue contribution is the square of the vertical component of the force over the cell. For the three cases the number of particles in the primordium was 200 and *v*_*gr*_ = 6σ/τ. (*X*_0_, *v*_*f*_) = (14.7σ, 35σ/τ), (20σ, 42σ/τ), and (10.6σ, 29σ/τ) for circular, oblate and prolate primordium, respectively.

A tentative explanation of this shape-dependent behavior could be found in the direction of the forces over the cells in the developing eye. In Figure 7 the orientation of mechanical forces on each cell, which have been calculated over the whole simulation (see methods) are represented for circular (*X*_0_ = *Y*_0_), oblate (*X*_0_ > *Y*_0_) and prolate (*X*_0_ < *Y*_0_) primordia. In these images the cells in the *A* region experiencing a net vertical force are colored blue, while those experiencing a net horizontal force are colored green. Cells in *P* region are red. In our simulation model, these forces are the result of the intercellular interactions. As cells grow, they push against each other, establishing the overall force distribution shown in Figure 7.

When comparing the situations with these different primordia, we observe that there are important differences in the force distribution. Thus, already in the initial stage, forces tend to have a radial orientation, without any preferred global direction in the case of the circular primordium. However, in the oblate primordium there is an over-representation of cells with vertical forces, while in the prolate primordium, horizontal forces dominate. As a consequence of this anisotropy in force distribution, cells will reorganize themselves locally so that the primordium will tend to become more circular as development proceeds. As a consequence, the prolate primordium will require faster differentiation speeds than expected for a circular primordium of equal *X*_0_ to terminate, as the former will tend to expand faster along its anterior-posterior axis. The opposite situation will occur with an oblate primordium. This also explains the fact that, for a given value of $\tilde{F}$, eyes with prolate primodium need more time than predicted by theory to finish their growth, reaching larger sizes, in the opposite direction than for oblate primordia, as shown in Figures 4, 5A.

Regardless of the shape of the primordia, throughout the growth of the eye the number of cells with horizontal forces increases. In Figure 7 it is observed how this orientation of the forces over cells (represented in this figure by the green color of the cells) is dominant in the advanced stages of development. This causes that, because we are not considering other sources of mechanical interaction such as surrounding epithelial tissue or other organs, in all three cases a tendency toward an oblate final shape is observed.

## 4. Discussion

In this article we have presented a theoretical model in an attempt to shed light on the dynamics and geometrical constrains of *Drosophila* eye growth, used here as a model for any developing system where growth and differentiation are coupled through a moving wave. The predictions of our model have been compared with the available experimental results. The first relevant point is the good agreement between theoretical predictions and experimental and simulation data, despite the simplicity of the model. Interestingly, our model does not include the area-dependence of the growth rate, suggesting that this decrease in growth rate with developmental time might have a modest role in controlling final eye size, although this decrease might have a significant effect in reducing developmental noise (i.e., variation in final eye size) as suggested by Vollmer et al. (2016).

The theoretical model finds some universal behaviors. Specifically, it predicts that both the time required for the eye to finalize growth, as well as the relationship between final and initial size of the eye depend, via two mathematical expressions, on a single parameter $\tilde{F}$, that governs the relationship between the width of the primordium, the rate of cell proliferation, and the velocity of the differentiation wave. These universal expressions show the relevance of geometric constraints in the development of the eye. A consequence of these universal laws is the existence of a limit value of $\tilde{F}$ for the completion of eye development, such that if $\tilde{F}<0.5$ eye growth does not terminate. In the vicinity of this value, our model predicts that small variations of $\tilde{F}$ would cause important changes in the final size of the eye, making the system more sensitive to noise. We have shown that, indeed, the genetically manipulated *GMR*>*Upd* strain, which lies in the sensitive region of parameter values, shows the greatest levels of eye size variability among the strains we have studied (Figure 5B). This result suggests that, in order to ensure robustness in organ size and developmental time, evolutionary pressure would have selected relationships between *v*_*f*_ and *v*_*g*_ away from these values.

The concordance between the predictions made by the theoretical and computational models, plus their agreement with experimental data, suggest that our theory captures sufficiently well the underlying biological process to serve as guide to biologists in making predictions about how variations in parameters such as differentiation speed (*v*_*f*_), growth rates (*k*) or geometry could explain eye size and its variation.

**Figure 8:**
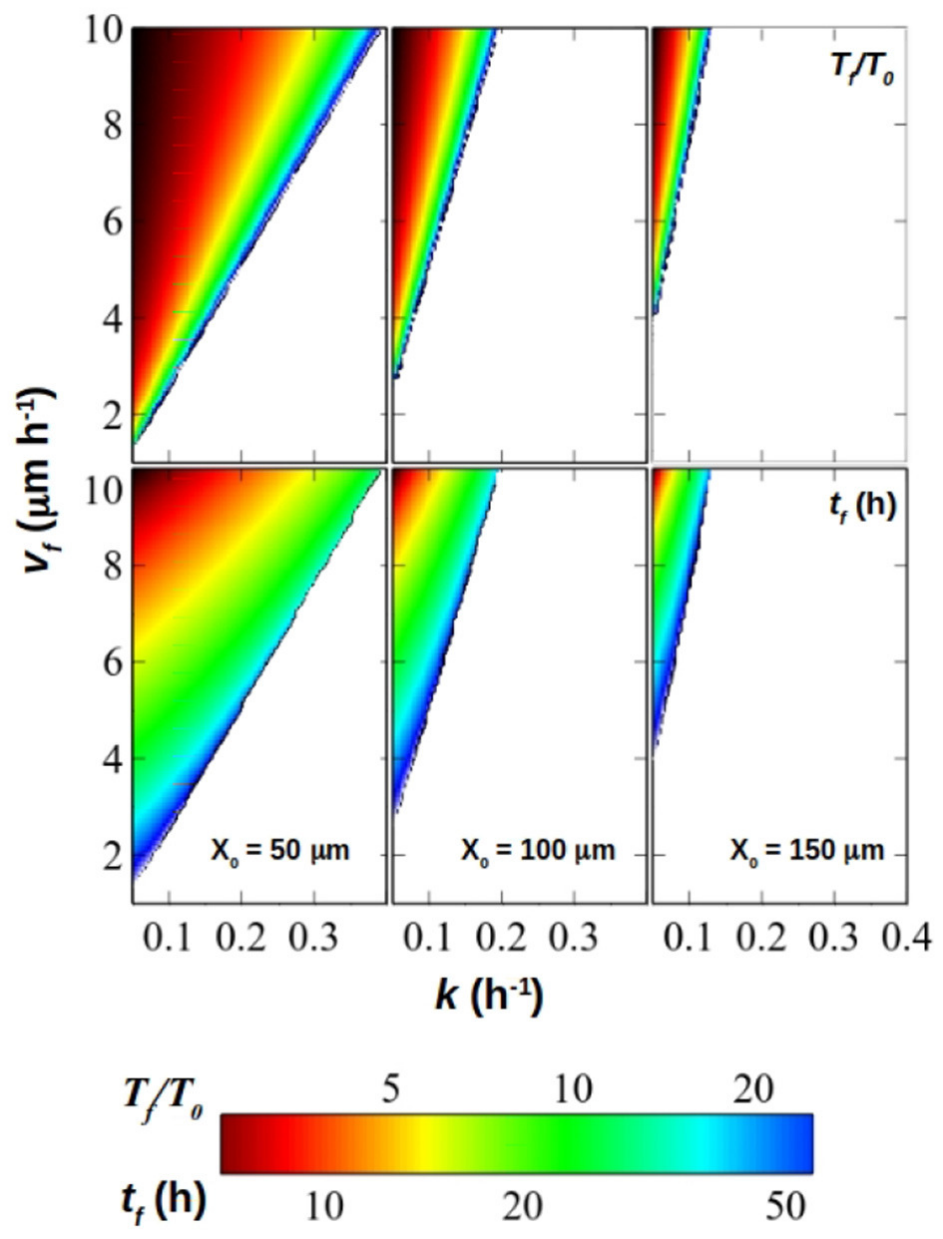
Relative final size of the eye (*T*_*f*_/*T*_0_, top panel) and eye termination time (*t*_*f*_, bottom panel) for primordium width *X*_0_ = 50μ*m* (left column), 100μ*m* (middle column) and 150μ*m* (right column) for values of *k* ∈ [0.05, 0.4]*h*^−1^ and $v_{f}\in \left[1,10\right]\mu mh^{-1}$ calculated with Equations (18, 17). The scale both for *T*_*f*_/*T*_0_ and *t*_*f*_ is display in the colorbar.

Figure 8 summarizes graphically these results. In it, the theoretical size of the eye's relative to its primordium (*T*_*f*_/*T*_0_) as well as the eye's developmental time *t*_*f*_ (i.e., time from the onset of differentiation of the primordium with *T*_0_ area and *X*_0_ width until it reaches *T*_*f*_, *t*_*f*_) are represented as a function of *v*_*f*_ and *k*, for a series of eye primordium widths (*X*_0_). This, together with Figures 4, 5A, help us predict different biological strategies for regulating eye size. For example, large eyes composed of a great number of ommatidia (and therefore of cells) are normally associated with an increased image resolution (Land, 1997). If the evolutionary goal is a large eye, which are the strategies that would ensure a developmentally robust eye size? The first strategy is typical of hemimetabolous insects, which include mayflies (*Ephemeroptera*), dragonflies (*Odonata*) or grasshoppers (*Orthoptera*), with species harboring very large eyes (Roonwal and Imms, 1947; Sherk, 1978; Friedrich and Benzer, 2000; Javier, 2016). In these animals, the first larval stage already has small functional eyes, which grow by adding one anterior strip of retinal tissue at each molt (sometimes several tens of them, such as in *Ephemeroptera*) until the adult size is attained. This mode of development, which is ancestral within insects, by being step-wise produces a small *T*_*f*_/*T*_0_ increase of eye size at each molt. This can be achieved with larger $\tilde{F}$, which, according to results in Figure 5A, fall within a “stable region”, that is, a region where variations of $\tilde{F}$ result in small variations in final size and which we deem as resilient in the face of environmental or developmental noise. In the grasshopper *Schistocerca*, the increase of eye area each molt is achieved by recruiting from a thin strip of progenitor cells (Friedrich, 2006), which should keep *X*_0_ small, contributing to larger $\tilde{F}$ values and therefore to maintain the system within the stable zone. In holometabolous insects, like flies, the eye develops at once within the last larval instar (and therefore, there are no intermediate molts), rather than growing step by step by accretion of strips of eye at each molt. For this type of development, which is exemplified by *Drosophila*, a first strategy would be to start off with a large eye primordium (i.e., large *T*_0_). In this case, *T*_*f*_/*T*_0_ would still be small, attainable with large $\tilde{F}$ values which would ensure a stable development. In this strategy, the eye primordium at the onset of differentiation should have grown to be large. However, the primordium should be narrow (small *X*_0_), in which case the final eye size could be also very narrow or, otherwise, it would demand very fast differentiation speeds (*v*_*f*_) and very slow division rates (*k*).

A second strategy would be to start with a small primordium, which would result in a large *T*_*f*_/*T*_0_ ratio. This strategy can be realized via smaller $\tilde{F}$, but toward the sensitive region. The data from *Drosophila melanogaster* strains (Vollmer et al., 2017b) indicates that *T*_*f*_/*T*_0_ ranges from 6 to 11, approximately. For the estimated values of *v*_*f*_ and *k*, and with *X*_0_ between 80*and* 120μ*m* approximately these strains lie along the curves in Figures 4, 5A on a region where variations in $\tilde{F}$ could result in significant changes in final size. As we have shown, the *GMR*>*Upd* strain in which a genetic perturbation affecting proliferation has been induced is the strain which lies further into the sensitive region. An effect of this is the major dispersion in adult eye size of the *GMR*>*Upd* strain when it is compared with the distribution of eye sizes in *GMR*>+ and *Or-R* strains. This predicted sensitivity for lower $\tilde{F}$ would be larger in species which, starting with a small primordium, develop very large eyes. Therefore, to maintain developmental stability (that is, to guarantee that final eye size does not vary despite of noise) the process of eye growth/differentiation should be provided with mechanisms to ensure a tight control of *v*_*f*_, *k* and *X*_0_, perhaps by means of feedback among them. In fact, it has been shown that Hedgehog (Hh) and Decapentaplegic (Dpp), two signaling molecules produced by the differentiating retina and required for the propagation of the differentiation wave, regulate the cell cycle of A cells in *Drosophila* (see, for example Firth and Baker, 2005). These results might indicate that the growth rate *k* and *v*_*f*_ might not be independent of each other. The role of Hh and Dpp is mostly of cell cycle synchronization (Baonza and Freeman, 2005; Lopes and Casares, 2010). Earlier experiments showing that Dpp acted as a general inhibitor of cell proliferation relied on overexpression and might not reflect its normal function (Penton et al., 1997). Still, flies harboring mutations that abolish this synchronization develop normal sized eyes (even though they accumulate some patterning defects; Mozer and Easwarachandran, 1999), indicating that the cell cycle synchronization exerted by Hh/Dpp does not have a general effect on the proliferation of progenitor cells. But even if *k* and *v*_*f*_ were interdependent, that would reduce the set of values that *k* and *v*_*f*_ could take. In other words, a situation in which *k* and *v*_*f*_ were mutually dependent would represent a special case within our analysis (i.e., it would be a subset of the solutions). However, this feedback could be stronger or weaker, or even non-existent, in other species. For example, the expression of the Dpp orthologues in *Schistocerca* and *Tribolium* suggests it plays a different role during eye development in these species (Friedrich and Benzer, 2000).

Interestingly, our model predicts, by combining (Equations 17, 18), that larger size increases from the primordium to the finalized eye will need longer developmental times, something that might only be possible through the coordination between the autonomous eye growth dynamics and the developmental time of the individual. Also, the time to termination would be more sensitive to variations in $\tilde{F}$ the smaller it is, demanding again mechanisms to allow adjusting the growth of the individual to potential variations in developmental time of the eye. In fact, such mechanisms have been recently discovered by which organs lagging behind relative to the rest of the organism send signals that delay development of the individual until their growth is completed (Garelli et al., 2012). Another interesting feature of eye development is that its size varies little with temperature Krafka (1920), within a range of viable temperature, despite the fact that developmental time shortens with increasing temperature (Al-Saffar et al., 1995). This could be achieved, according to our model, if *v*_*f*_ and *k* vary with temperature in the same degree—e.g., both parameters double if the culture temperature raises by, say, 10 degrees Celsius. If *X*_0_ did not vary, $\tilde{F}$ would remain the same, and would correspond to the same *t*_*f*_ · *k*. If *k* doubled, *t*_*f*_ would be halved and therefore the developmental time of the eye would match almost automatically the shortened individual's developmental time.

Despite the general agreement between theory and experimental data, which has allowed us to draw certain rules linking developmental variables to organ size, we investigated the role played by the shape of the primordium in the process, using our computational model. In this model, cells are subject to repulsive short range forces that avoid clumping and allow the tissue to maintain its 2D structure, akin normal epithelia. When we use this model to simulate eye development, we find a dependence of the final eye size with the primordium's shape, a dependence that is stronger the more elliptical the primordium is. This is clearly illustrated by simulation experiments, in which primordia of equal cell number grow to different sizes and taking different developmental times depending on the *Y*_0_/*X*_0_ ratio. We have explained the discrepancy between theory and simulation by the relevance of mechanical constrains in the development of the eye.

The final shape resembles the shape of the initial primordium, although we note a trend of generating eyes that are wider in the antero-posterior axis than in the dorsoventral axis (oblate). Although eye shape in *Diptera* is very variable (Casares and McGregor, 2021), as a general rule eyes tend to be prolate, with the dorsoventral axis being the longest. In *Drosophila*, the early eye primordium is prolate, and this general shape is maintained throughout development.

Although this is a prediction coming from a very simplified model, we believe that if unconstrained, a growing epithelium would adopt a circular conformation. The fact that the eye primordium, as it grows, maintains an elliptical shape might indicate that it is mechanically constrained. Indeed, the epithelium of the eye is in contact with the brain on its posterior side, connected to it through the optic nerve, and anteriorly with the antennal primordium (see Figure 1). Perhaps the interaction with these structures provides a mechanical constraint as the primordium grows. If this were the case, the regulation of the size and shape of the eye would depend also on these mechanical interactions. Interestingly, it has been recently shown that in some *Drosophila* species with larger eyes, the antennae are smaller (Ramaekers et al., 2019). Although the explanation for this phenomenon has been linked to temporal differences in gene expression, it is tantalizing to suggest that antennae of different size might also impact eye size by exerting different mechanical stress. Expanding our computational model to include adjacent tissues will help explore the potential mechanical influence on the final size and shape of the fly eye.

## Data Availability Statement

Data and software will be provided upon reasonable request to the corresponding author.

## Author Contributions

FC and AC contributed to the conception and design of the study. FL-C and AC developed the theory and programmed the codes, performed the simulations, and analyzed the data from simulations. TN and AI performed the experiments. FL-C, FC, and AC wrote the manuscript. All authors contributed to manuscript revision, read, and approved the submitted version.

## Conflict of Interest

The authors declare that the research was conducted in the absence of any commercial or financial relationships that could be construed as a potential conflict of interest.
